# Fluorination Induced Donor to Acceptor Transformation in A1–D–A2–D–A1-Type Photovoltaic Small Molecules

**DOI:** 10.3389/fchem.2018.00384

**Published:** 2018-08-30

**Authors:** Ruimin Zhou, Benzheng Xia, Huan Li, Zhen Wang, Yang Yang, Jianqi Zhang, Bo W. Laursen, Kun Lu, Zhixiang Wei

**Affiliations:** ^1^CAS Key Laboratory of Nanosystem and Hierarchical Fabrication, CAS Center for Excellence in Nanoscience, National Center for Nanoscience and Technology, Beijing, China; ^2^Sino-Danish College, University of Chinese Academy of Sciences, Beijing, China; ^3^Sino-Danish Center for Education and Research, Beijing, China; ^4^Nano-Science Center & Department of Chemistry, University of Copenhagen, Copenhagen, Denmark

**Keywords:** organic photovoltaics, small molecules, fluorination substitution, donors, acceptors

## Abstract

With the development of diversity of non-fullerene acceptors, it is found that there is no clear boundary between electron donors and electron acceptors. Modulation of the electron donating and withdrawing properties of organic semiconductors is necessary for organic photovoltaics research. In this work, we designed and synthesized three A1–D–A2–D–A1-type (A represents acceptor unit and D represents donor unit) small molecules, named as M-0F, M-1F, and M-2F, respectively containing zero, one, and two fluorine atoms in the terminal acceptor segments (A1), respectively. Fluorination substitution was found to be able to downshift the HOMO and LUMO energy level, red-shift the absorption, and enhance the electron mobility. The M-0F exhibited the highest efficiency of 5.99% as a donor in fullerene-containing system and the lowest efficiency of 0.58% as an acceptor in fullerene-free system. While the M-2F performed the lowest efficiency of 0.97% as the donor and the highest efficiency of 2.65% as the acceptor. The electron-donating and electron-withdrawing property of M-1F are in-between that of M-0F and M-2F. Among the three molecules, the electron mobility is increased while the hole mobility is decreased with increasing fluorination. This work provides a typical example of tuning of the electron donating and withdrawing property without changes to the backbone of the conjugated molecules, which is important for further designing high performance solution processable small molecules.

## Introduction

The bulk-heterojunction (BHJ) organic solar cells (OSCs) show a promising prospect for low-cost and renewable energy technology because of their unique advantages of light weight, easy-fabrication, and the capability to be fabricated into large area flexible devices (Forrest, 2004; Brabec et al., 2010; Kumar and Chand, 2012). In a typical BHJ organic photovoltaic (OPV) device, the heterojunction usually consists of a p-type electron donor and an n-type electron acceptor, which is the photoactive part for converting solar light to electricity. The p-type electron donor can be polymers or organic small molecules. The n-type electron acceptors include fullerene derivatives, such as [6, 6]-phenyl-C_61_/C_71_-butyric acid methyl ester (PC_61_BM/PC_71_BM) and non-fullerene electron acceptors. For a highly-efficient BHJ OPV, its active layer should possess the following features: (a) a broad absorption spectrum with a high extinction coefficient to utilize more solar photons; (b) a suitable molecular energy level alignment between the involved molecular orbitals on donor and acceptor to offer a sufficient driving force for efficient charge separation; (c) a bicontinuous network with nanoscale phase separation to facilitate exciton diffusion and charge separation; and (d) high charge mobility to facilitate charge transport (Scharber et al., 2006; Chen and Cao, 2009; Cheng et al., 2009; Beaujuge and Frechet, 2011; Henson et al., 2012; Li, 2012; Xu and Yu, 2014; Etxebarria et al., 2015; Zhang H. et al., 2015). Among these key features, the difference in LUMO energy of donor and acceptor is usually larger than 0.3 eV in fullerene system (Scharber and Sariciftci, 2013). However, with the development and application of non-fullerene electron acceptors, it is found that there is no clear boundary between electron donor and electron acceptor materials, and the difference of LUMO or HOMO energies in non-fullerene system could be very small. For instance, the polymer acceptor P-BNBP-fBT has a LUMO of −3.6 eV, and PTB7-Th donor has a LUMO level of −3.42 eV. The device based on PTB7-Th/P-BNBP-fBT showed a power conversation efficiency (PCE) as high as 6.26%, even though the difference in LUMO levels is only 0.18 eV (Long et al., 2016). However, polymer materials normally have a large polydispersity in molecular weight increasing the complexity of the materials. Thus, when it comes to fine tuning of the molecular levels, the use of solution processable small molecules is important to understand the relationship between molecular structures and their electron donating/withdrawing properties (Zhu et al., 2016; Wu et al., 2017).

At present, the small molecules could be designed as donor or acceptors based on different molecular architectures. For small molecule donors the conjugated skeleton is most often relatively planar. For example, oligothiophene-based small molecule donor DRCN7T exhibited an impressive optimized PCE of 9.30% using PC_71_BM acceptor (Zhang Q. et al., 2015). Our group synthesized the A1–D–A2–D–A1 structure electron-donating small molecule with a PCE of more than 9% with enhanced molecular planarity and crystallinity (Yuan et al., 2016). On the other hand, currently non-fullerene acceptors are mainly concentrated on fused ring acceptors (Cheng et al., 2018; Hou et al., 2018). The acceptors based on perylene diimide (PDI) or naphthalene diimide (NDI) have shown relatively good performance in PSCs (Facchetti, 2013; Zhang et al., 2013; Hartnett et al., 2014; Li et al., 2014; Lin et al., 2014; Jung et al., 2015; Liu et al., 2015; Sun et al., 2015; Yang et al., 2016; Lei et al., 2018). Most of the high-performance PDI and NDI-based fullerene-free acceptors have twisted backbones to decrease the planarity, self-aggregation, and crystalline domains of simple rylene diimides. Besides the rylene-based fullerene-free electron acceptors, electron-donating extended fused rings, e.g., indacenodithiophene (IDT) and indacenodithieno[3,2-b]thiophene (IDTT), were widely used in small molecule acceptors because their LUMO levels can be readily tuned by flanking with different electron-withdrawing groups and the steric effect of tetrahexylphenyl substituents on the coplanar backbone can reduce the intermolecular interactions while weaken the stacking of donor units and promote the stacking of acceptor units (Lin et al., 2015; Wu et al., 2015; Lin and Zhan, 2016; Huang et al., 2017; Yu et al., 2017). Another example was present by Hou et al., the steric hindrance caused by side chains could convert two isomers to donors and acceptors separately (Liu et al., 2018). In this case, molecules with weak π-π stacking structure is more likely to work as acceptor materials. Although recent progress showed that small molecules based on the same backbone can be changed from donor to accepters by changing the side chain hindrance, it is still unclear whether one can slightly tune the energy levels of planer small molecules and charge mobility to realize the transformation from donor to acceptor.

**Scheme 1:**
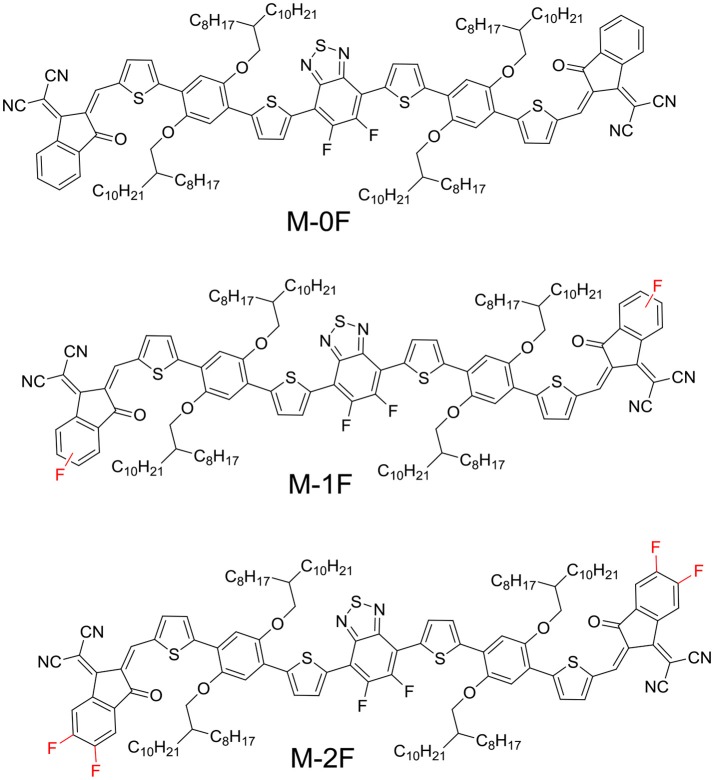
Chemical structures of M-0F, M-1F, M-2F molecules.

Herein, we designed and synthesized three novel A1–D–A2–D–A1-type small molecules with dialkoxyphenyldithiophene (PDT) as D unit, difluorinated benzothiadiazole (2FBT) as A2 unit and strong electron-withdraw group 1, 1-dicyanomethylene-3-indanone (IC) substituted with 0–2 fluorine substituents as end-capped A1, namely M-0F, M-1F, and M-2F. All three molecules exhibit good planarity. The molecular structures are shown in Scheme 1. Fluorine is the most electronegative element with relatively small van der Waals radius. It can serve as an electron-withdrawing group without introducing undesirable steric hindrance. The introduction of fluorine onto the conjugated backbone of polymers or small molecules for BHJ solar cells could lower the LUMO and HOMO level (Price et al., 2011; Zhou et al., 2011; Zhang et al., 2017; Zhao et al., 2017). Fluorination IC also promotes intermolecular interactions through forming non-covalent F–S and F–H bonds, which can be favorable for charge transport (Sakamoto et al., 2001; Lei et al., 2014; Kim et al., 2015). The target molecules show red-shifted absorption spectrum and their LUMO and HOMO energies decreased with increasing fluorination. As donor, M-0F exhibited the highest PCE of 5.99%, M-1F performed a PCE of 2.60%, and M-2F showed the lowest PCE of 0.97% with PC_71_BM as acceptor. On the other hand, as acceptor, M-0F showed the lowest PCE of 0.58%, M-1F performed medium PCE of 1.85%, and M-2F achieved the highest PCE of 2.65% with polymer PBDB-T as donor. The energy level difference affects the performance of the molecule to a certain extent, M-0F exhibited the same LUMO level as PBDB-T, and cannot provide enough driving force for charge transfer and separation, but for M-2F, its lower LUMO is relatively better-match with PBDB-T although the LUMO difference is small. On the other hand, the hole and electron mobility also show that transition from hole transport to electron transport can be obtained via fluorination.

## Results and discussion

### Molecular synthesis and properties

All three target molecules M-0F, M-1F, M-2F were synthesized according to the route shown in Scheme 1. To ensure the solubility of these three materials, the long branched side chain octyldodecyloxy was selected. Compound PDT2FBT-CHO was obtained through a Vilsmeier reaction from O-1. The three target molecules were synthesized by a Knoevenagel reaction. The synthetic routes, purification methods, and the nuclear magnetic resonance spectroscopy data are provided in the Supporting Information.

**Figure 1:**
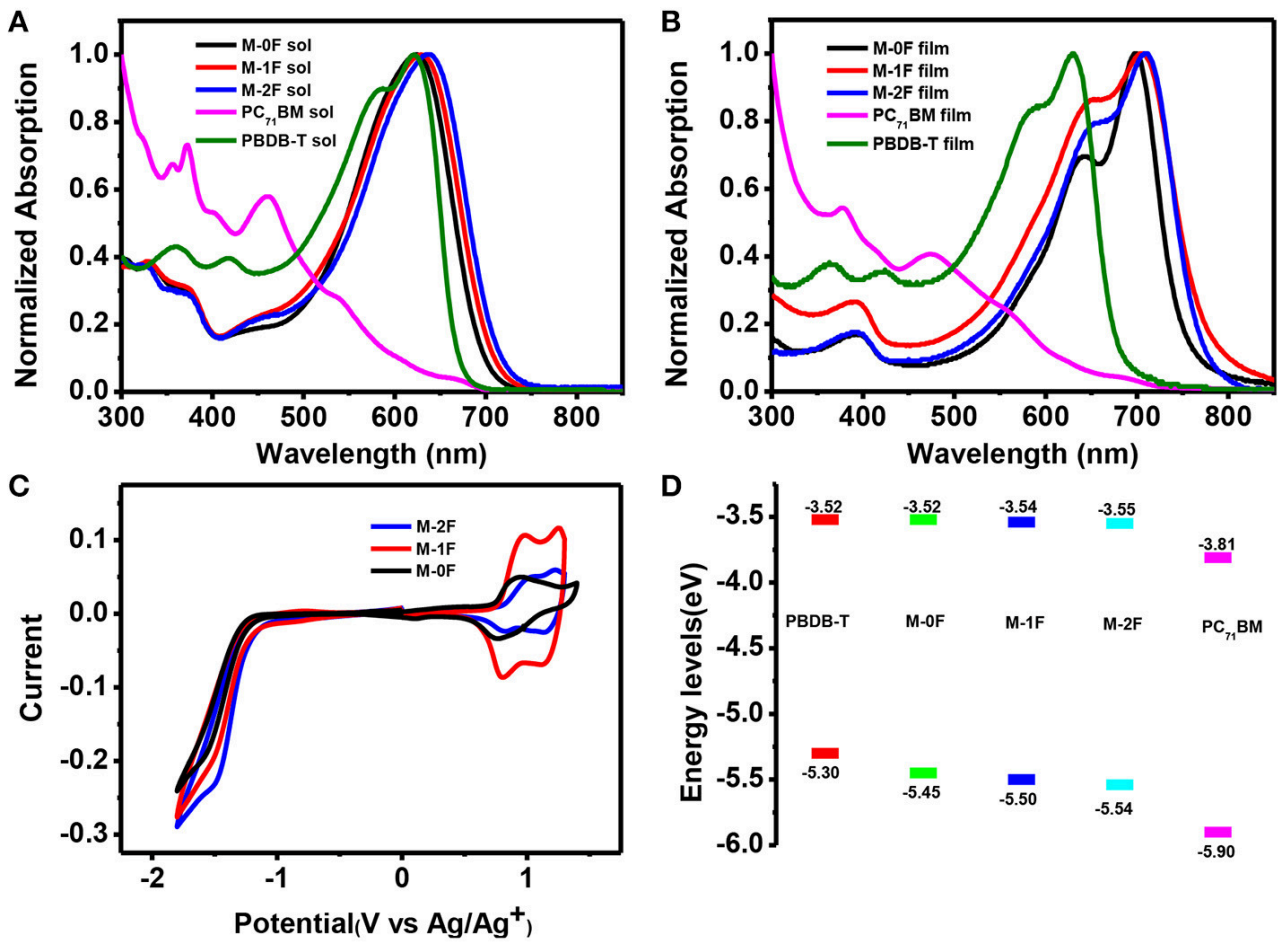
**(A)** The normalized UV-vis absorption spectra of M-0F, M-1F, and M-2F, PC_71_BM, PBDB-T in chloroform and **(B)** in thin film **(C)** Cyclic voltammograms of M-0F, M-1F, and M-2F **(D)** Energy level diagram of the related materials used in the device.

Figures 1A,B show the normalized UV–Vis absorption spectra of M-0F, M-1F, and M-2F measured in chloroform solution and in thin solid films. Detailed parameters are listed in Table 1. In solution, the three molecules show similar absorption spectra where the introduction of fluorine results in a red-shift of the absorption spectrum with the max absorption peak shifting from 623 to 630 and 638 nm. In thin films, all three molecules show more red-shifted and broader absorption spectra. The absorption peaks of these three compounds red-shift gradually from 697 to 705 nm and then to 712 nm in thin solid films. The strong shoulder peaks show that the three molecules have good π-π stacking in the film (Badgujar et al., 2016). The optical band gaps of M-0F, M-1F, and M-2F are calculated to be 1.64, 1.59, and 1.60 eV from the absorption edge, respectively.

**Table 1:** Optical and electrochemical data of compounds M1-0F, M1-1F, and M1-2F.

**Compound**	**${\lambda_{\max}^{\text{solution}}}_{\;}\;$ (nm)**	**$\lambda_{\max}^{\text{film}}$ (nm)**	**${\text{E}}_{\text{g},\;\text{film}\;}^{\text{opt}}$ (eV)**	**HOMO^CV^ (eV)**	**LUMO^CV^ (eV)**
M-0F	623	697	1.64	−5.45	−3.52
M-1F	630	705	1.59	−5.50	−3.54
M-2F	638	712	1.60	−5.54	−3.55

The cyclic voltammetry (CV) was used to evaluate the electrochemical properties of the three small molecules and the results are shown in Figures 1C,D with reversible reduction waves and quasi-reversible oxidation waves. The HOMO and LUMO energy levels are calculated from the onset oxidation and reduction potentials, assuming the absolute energy level of ${\text{FeCp}}_{2}^{+/0}$ to be 4.8 eV below vacuum. The equation of HOMO energy levels is E_HOMO_ = –e (Eox + 4.8–E_1/2_, (Fc/Fc^+^)) and the equation of the LUMO energy levels is E_LUMO_ = –e (E_RED_ + 4.8–E_1/2_, (Fc/Fc^+^)) (Li et al., 1999). Due to the strong electron withdraw ability of fluorine atom, the HOMO and LUMO of both M-1F, M-2F downshift. The HOMO levels of M-0F, M-1F, and M-2F are estimated to be −5.45, −5.50, and −5.54 eV and LUMO levels are −3.52, −3.54 to −3.55 eV (Figure 1D).

### Photovoltaic device characterization

**Figure 2:**
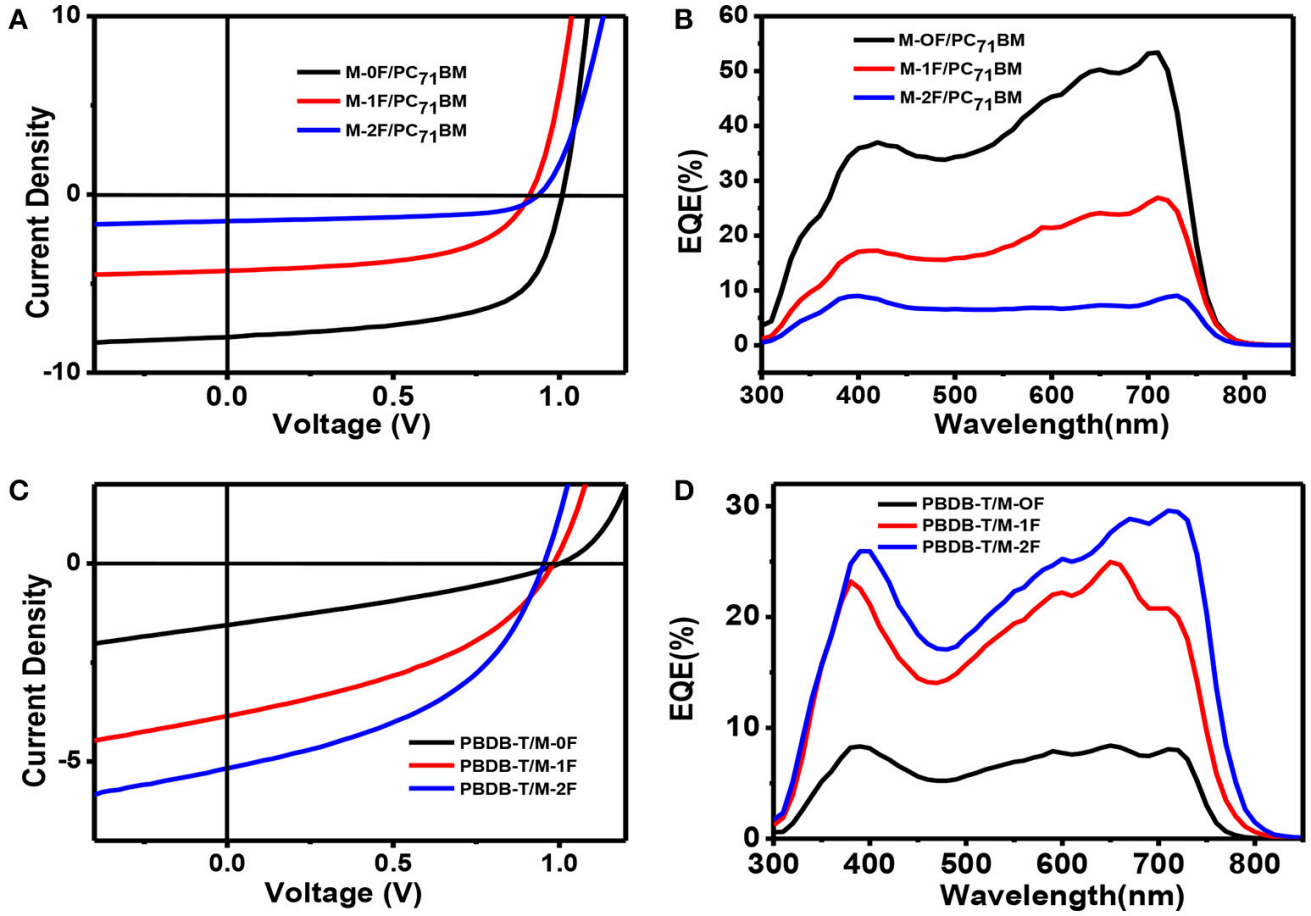
**(A,B)** J–V curves and EQE curves of M-0F, M-1F, M-2F With PC_71_BM blends at the best performance condition. **(C,D)** J–V curves and EQE curves of M-0F, M-1F, M-2F with PBDB-T blends at the best performance condition.

To investigate the photovoltaic behaviors of the three molecules as the electron donor and acceptor in OPV devices, we selected two materials, PC_71_BM as an acceptor (Wienk et al., 2003; Thompson and Fréchet, 2008) and PBDB-T as a donor polymer (Zhao et al., 2016) to blend with M-0F, M-1F, M-2F to fabricated conventional device structure: ITO/PEDOT:PSS/Donor:Acceptor/Ca/Al. We comparatively studied the photovoltaic performance of M-0F, M-1F, and M-2F as donor materials. The devices were made of blend of small molecule donor and PC_71_BM with chloroform as solvent by spin-coating. The photovoltaic properties of the devices were characterized under illumination of simulated solar light, AM1.5G (100 mWcm^−2^). The optimized current density vs. voltage (J-V) curves and their corresponding external quantum efficiency (EQE) curves are displayed in Figure 2. The corresponding photovoltaic performance data is summarized in Table 2. The photovoltaic performance data of other condition is summarized in Tables S1, S2. The device based on M-0F: PC_71_BM exhibited a high *V*_*OC*_ of 1.01 V, a *J*_*SC*_ of 9.54 mAcm^−2^ and a high FF of 62.41%, resulting in the highest optimal PCE of 5.99%. For device M-1F: PC_71_BM, PCE was 2.60% with *V*_*OC*_ of 0.91 V, *J*_*SC*_ of 5.14 mA/cm^2^ and FF 55.71%. However, for device M-2F: PC_71_BM, the PCE was only 0.97% with a *V*_*OC*_ of 0.93 V, a *J*_*SC*_ of 1.80 mAcm^−2^ and a FF of 57.65%. It can be concluded that as an electron donor in blends with PC_71_BM, M-0F displays a superior photovoltaic performance compared to M-1F while M-1F has better photovoltaic performance than M-2F. The results show that the device performance based the three molecules as donors decreases with increased number of fluorine atoms attached to the terminal acceptor units (A1).

**Table 2:** Device performance of M-0F, M-1F, and M-2F with PC_71_BM as acceptors.

**Donor/Acceptor**	***V_*OC*_*(V)**	***J_*SC*_*(mAcm^−2^)**	***FF* (%)**	**PCE (%)**	**Additive**
M-0F/PC_71_BM	1.01	9.54	62.41	5.99	1%DIO
M-1F/PC_71_BM	0.91	5.14	55.71	2.60	1.5%DIO
M-2F/PC_71_BM	0.93	1.80	57.65	0.97	1.5%DIO
PBDB-T/ M-0F	0.99	1.88	30.95	0.58	1%CN
PBDB-T/ M-1F	0.98	4.62	40.84	1.85	1%CN
PBDB-T/ M-2F	0.95	6.25	44.54	2.65	1%CN

We also studied photovoltaic performance of M-0F, M-1F, and M-2F as electron acceptor material with PBDB-T as donor material. The blend of small molecule acceptor and PBDB-T was made with chlorobenzene as solvent by spin-coating. It was found that the PCE of a M-0F:PBDB-T device was only 0.58% with low *J*_*SC*_ and FF; the PCE of a device based on M-1F:PBDB-T was 1.85%; while the device based on M-2F:PBDB-T displayed a PCE as high as 2.65%, with a *V*_*OC*_ of 0.95, a *J*_*SC*_ of 6.25 mAcm^−2^, and a FF of 44.54%. It can be concluded that, as electron acceptors after blending with PBDB-T, M-2F displays a superior photovoltaic performance compared to M-0F and M-1F. This means, that as acceptors and donors the series of small molecules display opposite trend in device performances. With increasing fluorination, the device performance as donor decreases while improves as acceptor.

### Photoluminescence quenching effects

In order to investigate the donor property of M-0F, M-1F, and M-2F, the fluorescence spectra were measured in the range of 680–900 nm with exciting wavelength at 650 nm. As shown in Figure S1A, the photoluminescence (PL) of M-0F, M-1F can be partly quenched by PC_71_BM, whereas the PL of M-2F almost can't be quenched by PC_71_BM, indicating that the exciton dissociation in the blend of M-0F/PC_71_BM, M-1F/PC_71_BM should be more efficient than that in the blend of M-2F/PC_71_BM. For studying acceptor property of these three small molecules, the exciting wavelength at 740 nm was chosen as shown in Figure S1B. From the film of M-0F/ PBDB-T, M-1F/ PBDB-T to M-2F/ PBDB-T, the PL quenching effect is gradually stronger, indicating that the exciton dissociation is more efficient gradually. These results are in consistent with device performance.

### Film morphology and microstructure

**Figure 3:**
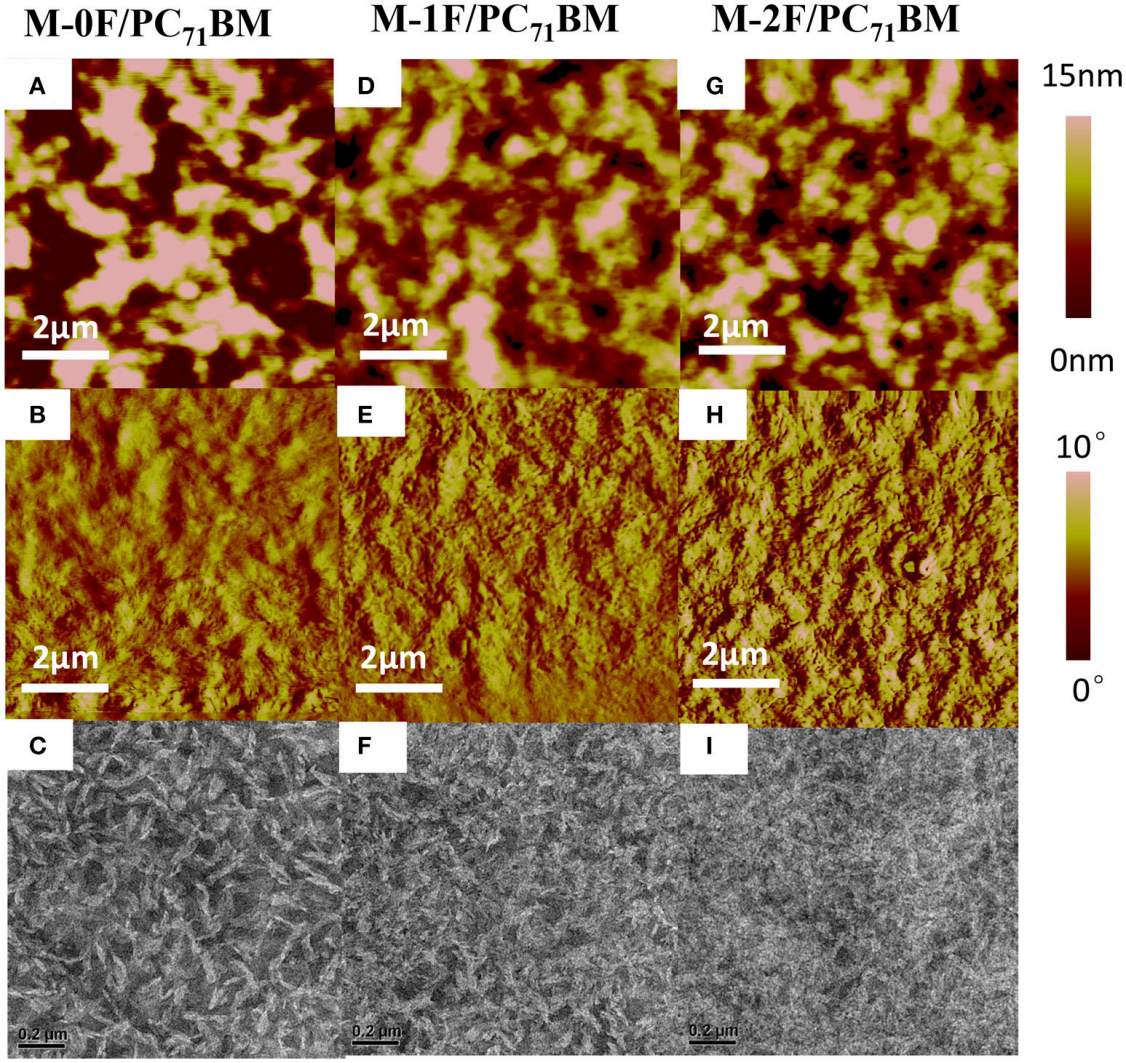
Tapping-mode AFM **(A,D,G)** topography and **(B,E,H)** phase images and **(C,F,I)** TEM images of **(A–C)** M-0F/PC_71_BM, **(D–F)** M-1F/PC_71_BM, **(G–I)** M-2F/PC_71_BM blend films.

Using atomic force microscopy (AFM) and transmission electronic microscopy (TEM), the phase separation morphology of the blends for the two systems were investigated. As shown in Figure 3, the AFM images are consistent with the TEM images. For the PC_71_BM system, it can be seen that M-0F, M-1F, and M-2F have good compatibility with PC_71_BM and good phase separation. The surface roughness is 8.317, 4.236, and 4.617 nm for blend films M-0F:PC_71_BM, M-1F:PC_71_BM, and M-2F:PC_71_BM, respectively. The M-0F:PC_71_BM blend has the larger size of aggregation phase region, and the proper domain size for efficient exciton diffusion and dissociation contributing to high *J*_*SC*_ and FF. For M-1F:PC_71_BM and M-2F:PC_71_BM blend film, the aggregation size is so small resulting in low *J*_*SC*_ and FF.

**Figure 4:**
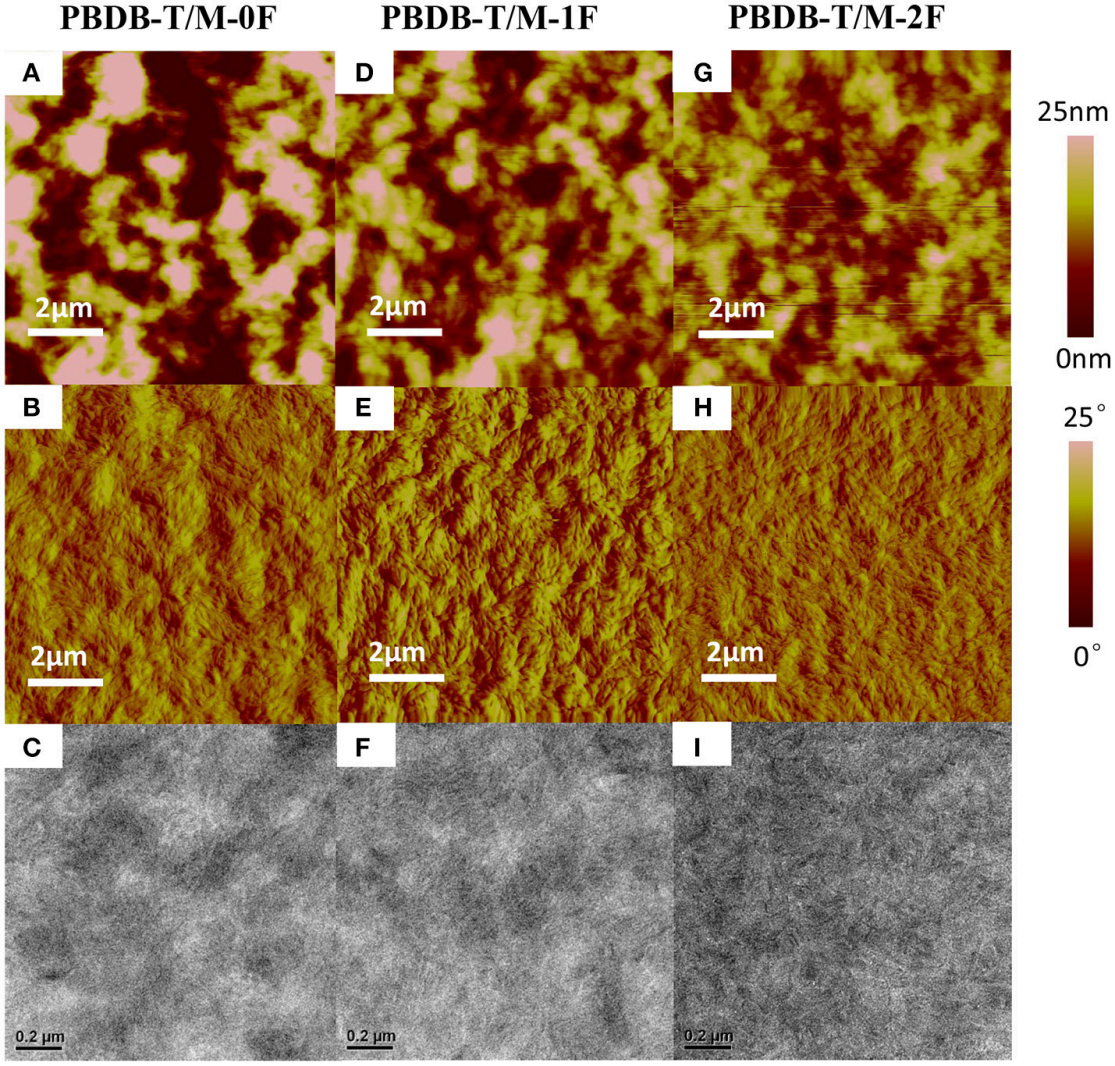
Tapping-mode AFM **(A,D,G)** topography and **(B,E,H)** phase images and **(C,F,I)** TEM images of **(A–C)** PBDB-T/M-0F, **(D–F)** PBDB-T/M-1F, **(G–I)** PBDB-T/M-2F blend films.

For the non-fullerene system shown in Figure 4, the surface roughness of PBDB-T:M-0F, PBDB-T:M-1F, and PBDB-T:M-2F blend film are 11.034, 6.672, and 4.356 nm. The surface of PBDB-T:M-2F is smoother. From TEM image, we can see that PBDB-T:M-2F formed a uniform nanoscale phase separation with nanofiber and continuous interpenetrating network structure, and the phase separation size is suitable, which facilitates the exciton diffusion and charge transfer and improve *J*_*SC*_ and FF. However, For the PBDB-T:M-0F and PBDB-T:M-1F blends large domain size can be seen, this is not conducive to the charge transfer, which cause a negative impact on the *J*_*SC*_ and FF.

**Figure 5:**
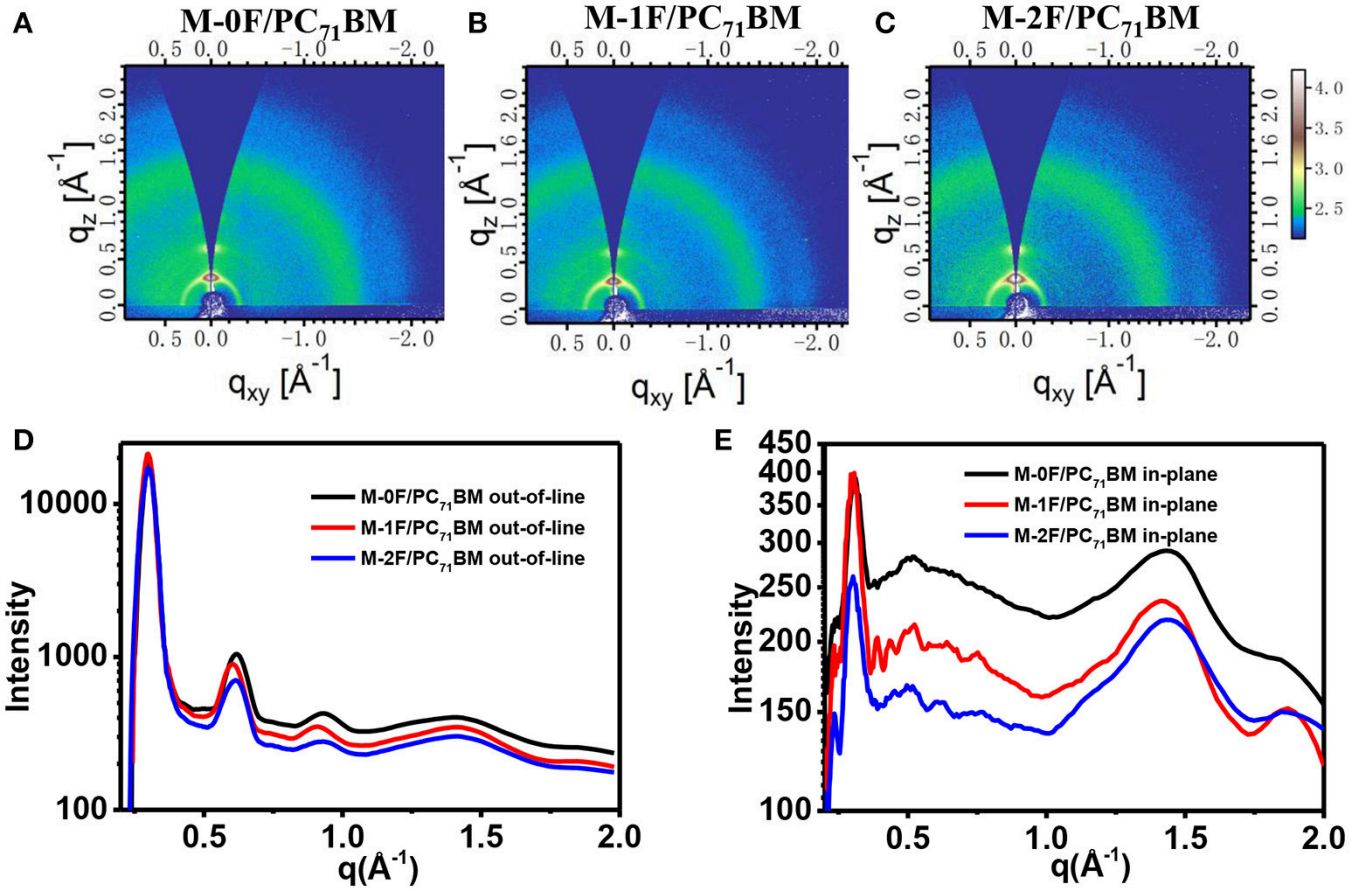
2D GIWAXS patterns of M-0F/PC_71_BM **(A)**, M-1F/PC_71_BM **(B)**, and M-2F/PC_71_BM **(C)** blend film. Out-of-plane **(D)** and in-plane **(E)** cuts of the corresponding 2D GIWAXS patterns.

In order to further study the molecular stacking and crystallization properties of the active layer, grazing incidence wide-angle X-ray scattering (GIWAXS) on the neat films and blend films was employed. The results of pristine M-0F, M-1F, M-2F, and PBDB-T films are shown in Figure S2. In-plane direction M-0F, M-1F, and M-2F all show π-π stacking peaks at 1.72 Å^−1^ (d-spacing: 3.65 Å), 1.77 Å^−1^ (d-spacing: 3.55 Å), 1.78 Å^−1^ (d-spacing: 3.53 Å), which indicates the higher crystallinity of M-1F, and M-2F compared with M-0F in the neat films. Figure 5 shows the two-dimensional GIWAXS patterns and the one-dimensional GIWAXS cuts along in-plane and out-of-plane directions of M-0F:PC_71_BM, M-1F:PC_71_BM, and M-2F:PC_71_BM blended films. As can be seen from Figure 5, M-0F, M-1F, and M-2F all show very obvious diffraction peaks of (100), (200), (300), and (010), indicating the good crystallinity M-0F, M-1F, and M-2F with the orderly aggregation state structure. Besides, these corresponding peaks have almost the same location, which indicate the same d-spacing. The positions of (100) peak is ~0.298 Å^−1^ correspongding to a d-spacing of ~21.08 Å. The positions of (010) q is ~1.869 Å^−1^, so the d-spacing is ~3.36 Å. The π-π stacking of M-1F is stronger while the hole mobility is not the highest compared with M-0F, M-2F, indicating that the performance of the donor and acceptor has been changed.

**Figure 6:**
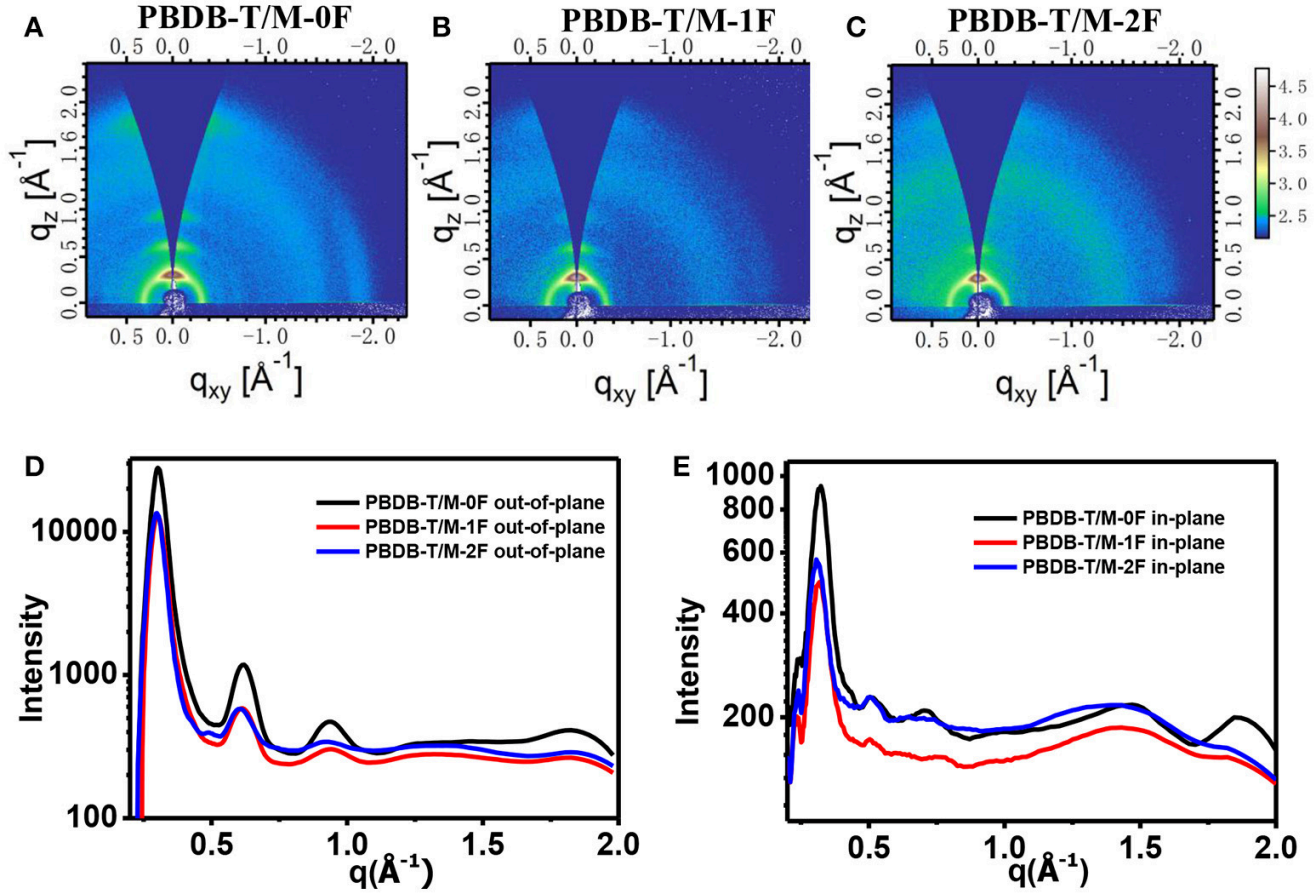
2D GIWAXS patterns of PBDB-T/ M-0F **(A)**, PBDB-T/ M-1F **(B)**, and PBDB-T/ M-2F **(C)** blend film. Out-of-plane **(D)** and in-plane **(E)** cuts of the corresponding 2D GIWAXS patterns.

Figure 6 shows the GIWAXS of PBDB-T:M-0F, PBDB-T:M-1F, and PBDB-T:M-2F blend films. Similar with PC_71_BM system, M-0F, M-1F, and M-2F all show very obvious diffraction peaks of (100) (200), (300), and (010), these corresponding peaks have almost the same location. The positions of (100) q is~ 0.30 Å^−1^, so the d-spacing is ~20.94 Å. The positions of (010) q is ~1.83 Å^−1^, so the d-spacing is ~3.43 Å. The GIWAXS results show the good crystallization of M-0F, M-1F, and M-2F.

### Hole and electron mobility

In order to study the origin of the observed differences in photovoltaic properties of the three new materials, the hole and electron mobility of the three pure materials and blend films at the best performance condition were tested using space-limited charge (SCLC) method (Blom et al., 2005). The hole and electron mobility curves measured by the SCLC method are shown in Supplementary Information Figure S3. The data of hole and electron mobility under different conditions is summarized in Table 3. The hole and electron mobility for pristine M-0F are 2.49 × 10^−4^ and 1.01 × 10^−6^ cm^2^ V^−1^ S^−1^, respectively. For pristine M-1F, the hole and electron mobility are 1.78 × 10^−5^ and 3.03 × 10^−6^ cm^2^ V^−1^ S^−1^. For pristine M-2F, the hole and electron mobility are 6.42 × 10^−6^ and 1.26 × 10^−5^ cm^2^ V^−1^ S^−1^. This indicates the transition from hole transport to electron transport. Among M-1F and M-2F, the electron mobility of M-2F is higher than its hole mobility, so M-2F has better n-type semiconductor property than M-1F. At the corresponding best performance condition, from M-0F:PC_71_BM, M-1F:PC_71_BM to M-2F:PC_71_BM, both the hole and electron mobility decrease. From PBDB-T:M-0F, PBDB-T:M-1F to PBDB-T:M-2F, both the hole and electron mobility increase, which is consistent with photovoltaic performance results.

**Table 3:** SCLC measured hole mobility and electron mobility for pure M-0F, M-1F, M-2F, and their PC_71_BM or PBDB-T blends.

	**M-0F**	**M-1F**	**M-2F**	**M-0F /PC_71_BM**	**M-1F /PC_71_BM**	**M-2F /PC_71_BM**	**PBDB-T /M-0F**	**PBDB-T /M-1F**	**PBDB-T /M-2F**
Hole mobility (cm^2^ V^−1^ S^−1^)	2.49 × 10^−4^	1.78 × 10^−5^	6.42 × 10^−6^	9.26 × 10^−5^	3.90 × 10^−6^	1.74 × 10^−6^	9.25 × 10^−6^	1.47 × 10^−5^	6.87 × 10^−5^
Electron mobility (cm^2^ V^−1^ S^−1^)	1.01 × 10^−6^	3.03 × 10^−6^	1.26 × 10^−5^	5.20 × 10^−5^	3.84 × 10^−5^	1.52 × 10^−5^	4.27 × 10^−7^	2.07 × 10^−6^	3.7 × 10^−6^

On the basis of above observations, we can conclude that M-0F has good donor property with better phase separation and higher hole mobility in fullerene system. while M-2F has the best acceptor properties with nanofiber interpenetrating network morphology and higher electron mobility in non-fullerene system.

## Conclusion

In conclusion, we designed and synthesized three novel A1-D-A2-D-A1 small molecules (M-0F, M-1F, and M-2F) with the strong electron-withdraw group 1,1-dicyanomethylene-3-indanone (IC) substituted with varying number of fluorine atoms. With increasing number of fluorine atoms, the absorption spectra are red-shifted, and both the LUMO and HOMO energies are decreased. M-0F exhibited excellent electron-donating properties while M-2F showed excellent electron-accepting properties. Our work further proves that the definition of donor and acceptor is without clear boundaries, which offer wide potential for molecular design. The regulation of energy levels and carrier mobility is one of the effective ways to achieve this transformation. Also, the boundary between donor materials and acceptor materials is worth exploring for understanding the deep mechanism of exciton dissociation and charge transfer in BHJ active layers.

## Author contributions

RZ designed the project, finished the synthesis, characterization, wrote the first draft of the manuscript; BX designed the project, finished the device, and characterization of photovoltaic performance. BL, KL, and ZW guided the project and helped to revise the manuscript. The other authors gave the contribution in synthesis or data analysis.

### Conflict of interest statement

The authors declare that the research was conducted in the absence of any commercial or financial relationships that could be construed as a potential conflict of interest.
