# Supplementary material for: Fluorination Induced Donor to Acceptor Transformation in A1–D–A2–D–A1-Type Photovoltaic Small Molecules
Source: Front Chem. 2018 Aug 30;6:384. doi: 10.3389/fchem.2018.00384 (PMC6127638; doi:10.3389/fchem.2018.00384)

Supplementary Material

**Fluorination Induced Donor to Acceptor Transformation in A1–D–A2–D–A1-type Photovoltaic Small Molecules**

**Ruimin Zhou^1,2,3,4#^, Benzheng Xia^1#^, Huan Li^1^, Zhen Wang^1^, Yang Yang^1^, Jianqi Zhang^1^, Bo W. Laursen^4^, Kun Lu^1^* and Zhixiang Wei^1^***

^1^ CAS Key Laboratory of Nanosystem and Hierarchical Fabrication, CAS Center for Excellence in Nanoscience, National Center for Nanoscience and Technology, Beijing, China.

^2^ Sino-Danish College, University of Chinese Academy of Sciences, Beijing, China

^3^ Sino-Danish Center for Education and Research, Beijing, China

^4^ Nano-Science Center & Department of Chemistry, University of Copenhagen, Copenhagen, Denmark

**Correspondence:** Corresponding Author: Kun Lu [lvk@nanoctr.cn](mailto:lvk@nanoctr.cn)Zhixiang Wei [weizx@nanoctr.cn](mailto:weizx@nanoctr.cn)

#: join the co-first authors

1. **Characterization**^1^H nuclear magnetic resonance (NMR) spectra were obtained on a Bruker DMX-400 NMR Spectrometer (operating at 400 MHz, using CDCl_3_ as solvent). The chemical shifts were calibrated using solvent peak of tetramethylsilane as reference for ^1^H-NMR and MALDI-TOF mass spectra (MS) were recorded on a Micromass GCT-MS spectrometer. UV–vis absorption spectra were obtained with a JASCO V-570 spectrophotometer. Cyclic voltammetry (CV) measurement was conducted on an electrochemical workstation (VMP3 Biologic, France) with Pt disk coated with M-0F, M-1F, M-2F films, Pt plate, and Ag/Ag+ electrode as working electrode, counter electrode and reference electrode respectively, in a 0.1 mol L^-1^ tetrabutylammonium phosphorus hexafluoride (Bu_4_NPF_6_) in acetonitrile solution. Redox potentials were internally calibrated using the ferrocene/ferrocenium (Fc/Fc+) redox couple (-4.8eV). Atomic force microscopy (AFM) images of the blend films were obtained from the devices directly on a VEECO Dimension 3100 atomic force microscope working under tapping mode. Transmission electron microscopy (TEM) images were obtained on a Tecnai G2 F20 U-TWIN TEM instrument. Films spin coated the same condition for solar cell devices on ITO substrates were immersed in water, and the floating active layers were transferred to TEM grid. Grazing-incidence Wide-Angle X-ray Scattering (GIWAXS) measurements were conducted on a Xenocs-SAXS/WAXS system with X-ray wavelength of 1.5418 Å. The film samples were irradiated at a fixed angle of 0.2^o^. All film samples are prepared by spin-coating chloroform or chlorobenzene solutions on PEDOT:PSS/ITO/glass substrates.
2. **Solar Cell Fabrication and Measurement**

ITO coated glass substrates were cleaned in deionized water, acetone and isopropyl alcohol for 10 min sequentially in ultrasonic bath. After dried in nitrogen flow, the substrates were subjected to UV/ozone treatment for 15 min. Then a PEDOT:PSS (3500 rpm, ~30 nm) layer was spin coated onto the substrate, and dried at 150 °C for 15 min in air. The substrates were transferred into a glove-box with nitrogen atmosphere. Subsequently, M-0F:PC_71_BM, M-1F:PC_71_BM and M-2F:PC_71_BM in a 10 mg mL^-1^ chloroform solution were spin-coated at 2000 rpm for 30 s to obtain a film thickness of approximately 100 nm. The blends of M-0F:PBDB-T, M-1F:PBDB-T and M-2F:PBDB-T at different D/A ratios were dissolved in solvent CB at a donor weight concentration of 10 mg mL^-1^. The thickness of the photovoltaic layer was ~100 nm, followed by deposition of the Ca/Al (20 nm/100 nm) cathode at a pressure of 2 × 10^-6^ Torr through a shadow mask. Active area of the cells is 0.04 cm^2^. The current density-voltage (J-V) curves were obtained by a Keithley 2420 Source-Measure Unit. The photocurrent was measured under illumination using an Oriel Newport 150W Solar simulator (AM 1.5G). The EQE measurements of the devices were performed with an Oriel Newport System (Model 66902). All the measurements were performed at room temperature in air.

1. **Mobility measurement.**

All procedures are the same for solar cell devices, except for using Au (100 nm) to replace Ca/Al (20 nm/100 nm) cathode. The current density-voltage (*J-V*) curves in the range of 0-5 V were obtained by a Keithley 2420 Source-Measure Unit in the dark in the air Hole mobility can be calculated by fitting the results in the equation:
 𝐽 = 9ε_0_ε_r_μ𝑉^2^/8L^3^

where *J* is the current density, L is the film thickness of active layer, μ is the hole mobility, ε_r_ is the relative dielectric constant of the transport medium. It is assumed to be 3, which is typical for conjugated organic materials. ε_0_ is the permittivity of free space, V is the internal voltage in the device and *V* = *V*_appl_ - *V*_r_ - *V*_bi_, where *V*_appl_ is the applied voltage to the device, *V*_r_ is the voltage drop due to contact resistance and series resistance across the electrodes, and *V*_bi_ is the built-in voltage due to the relative work function difference of the two electrodes.

1. **Materials and synthesis**

Scheme S1 The synthetic routes of the target molecules

Unless stated otherwise, all the solvents and chemical reagents used were obtained commercially and were used without further purification. Drying of pyridine, chloroform, 1,2-dichloroethane: Add anhydrous calcium chloride for 3 days, filter with a dry glass funnel and a conical flask , collect the solvent in a dried reagent bottle and add the muffle-dried A4 molecular sieve. Compounds O-1 was purchased from Solar Materials Inc. (Beijing). Compound 1-3 were purchased from **Derthon Optoelectronic Materials Science Technology Co LTD.**

**Compound PDT2FBT-CHO.** A solution of **O-1** (2000 mg, 1.09 mmol) in 50 mL anhydrous 1,2-dichloroethane and anhydrous DMF (0.34 mL, 4.36 mmol) was cooled down to 0 ^o^C under nitrogen atmosphere. POCl_3_ (0.42 mL, 4.36 mmol) was added dropwise into the solution within 10 min. The mixture was stirred at 0 ^o^C for 30 min and room temperature for 1 hour respectively. Then it was heated to refluxing at 85 ^o^C for 16 hours. The solution was cooled down to room temperature and 40 mL sodium acetate solution (1 M in water) was added. The mixed solution was stirred for 30 min at room temperature. Then it was poured into water, extracted with dichloromethane, dried with MgSO_4_ and concentrated under reduced pressure. The crude product was purified by silica gel chromatography with dichloromethane/petroleum ether (1: 1) as eluent, affording **3** as deep red solid. ^1^H-NMR (400 MHz, CDCl3) δ 9.83 (s, 2H), 8.31 (d, J = 3.5 Hz, 2H), 7.66 (d, J = 3.9 Hz, 2H), 7.60 (d, J = 3.6 Hz, 2H), , 7.28(s, 2H), 7.25 (s, 2H), 4.05 (d, J = 4.8 Hz, 8H), 2.04 – 1.96 (m, 4H), 1.71– 1.24(m, 128H), 0.91– 0.84 (m, 24H). m/z (MALDI-TOF) 1893.23 calcd. for C_116_H_178_F_2_N_2_O_6_S_5_1894.

**Compound M-0F.** To a solution of **PDT2FBT-CHO** (400mg, 0.211 mmol) and indene-1, 3-dione (616mg, 4.22mmol) in 50 mL anhydrous chloroform was added 3 drops of triethylamine. The mixture was stirred in nitrogen atmosphere for 24 hours at room temperature. Then the mixture was precipitated in methanol to get a blue solid. The crude product was further purified by silica gel chromatography with chloroform/petroleum ether (3: 1) as eluent, affording **M-OF** as blue solid. ^1^H-NMR (400 MHz, CDCl3) δ 8.79 (s, 2H), 8.66 – 8.61 (m, 2H), 8.32 (d, J = 4.0 Hz, 2H), 7.91 (dd, J = 5.5, 2.7 Hz, 4H), 7.74 (dd, J = 8.8, 4.9 Hz, 4H), 7.68 (dd, J = 9.6, 4.2 Hz, 4H), 4.12 (s, 8H). 2.13-2.05(m, 4H), 1.4-1.15(m, 128H), 0.95-0.8(m, 24H). m/z (MALDI-TOF) 2244 calcd. for C_140_H_186_F_2_N_6_O_6_S_5_ 2245.3.

**Compound M-1F.** To a solution of **PDT2FBT-CHO** (400mg, 0.21 mmol) and 2-(3-oxo-2,3-dihydro-1H-inden-1-ylidene)malononitrile compound with fluoromethane (1:1) (957 mg, 4.22 mmol) in 50 mL anhydrous chloroform was added 3 drops of triethylamine. The mixture was stirred in nitrogen atmosphere for 24 hours at room temperature. Then the mixture was precipitated in methanol to get a blue solid. The crude product was further purified by silica gel chromatography with chloroform/petroleum ether (4: 1) as eluent, affording **M-1** as blue solid. 1H NMR (400 MHz, CDCl3) δ 8.85 (d, J = 3.2 Hz, 2H), 8.69 (dd, J = 8.7, 4.3 Hz, 1H), 8.36 (dd, J = 9.0, 1.9 Hz, 3H), 7.99 – 7.91 (m, 3H), 7.78 – 7.69 (m, 4H), 7.56 (dd, J = 7.5, 3.3 Hz, 1H), 7.47 – 7.40 (m, 2H), 7.36 (s, 4H), 4.14 (s, 8H), 2.16 – 2.01 (m, 4H), 1.78 – 1.14 (m, 128H), 0.93 – 0.81 (m, 24H). m/z (MALDI-TOF) 2312 calcd. for C_142_H_192_F_4_N_6_O_6_S_5_ 2313.

**Compound M-2F.** To a solution of **PDT2FBT-CHO** (400 mg, 0.21 mmol) and 2-(5,6-difluoro-3-oxo-2,3-dihydro-1H-inden-1-ylidene)malononitrile (966 mg, 4.2 mmol) in 50 mL anhydrous chloroform was added 3 drops of triethylamine. The mixture was stirred in nitrogen atmosphere for 24 hours at room temperature. Then the mixture was precipitated in methanol to get a blue solid. The crude product was further purified by silica gel chromatography with chloroform/petroleum ether (4: 1) as eluent, affording **M-2F** as blue solid. ^1^H NMR (400 MHz, CDCl3) δ 8.78 (s, 2H), 8.46 (dd, J = 9.9, 6.6 Hz, 2H), 8.32 (s, 2H), 7.92 (s, 2H), 7.71 (s, 2H), 7.66 (t, J = 7.5 Hz, 4H), 4.13 (s, 8H), 2.18–2.00 (m, 4H), 1.56–1.15 (m, 128H), 0.86 (dt, J = 7.2, 4.6 Hz, 24H). m/z (MALDI-TOF) 2316 calcd. for C_140_H_182_F_6_N_6_O_6_S_5_ 2317.2.

**Table S1.** Device performance of M-0F, M-1F and M-2F with PC_71_BM as acceptors

| Donor/Acceptor | *Voc*  (V) | *J_sc_*  (mA/cm^-2^) | *FF*  (%) | *PCE*  (%) | additive |
| --- | --- | --- | --- | --- | --- |
| M-0F/ PC_71_BM | 1.01 | 9.54 | 62.41 | 5.99 | 1%DIO |
| M-0F/ PC_71_BM | 0.91 | 9.65 | 59.40 | 5.23 | 1.25%DIO |
| M-0F/ PC_71_BM | 0.99 | 7.64 | 62.72 | 4.79 | 1.5%DIO |
| M-0F/ PC_71_BM | 0.97 | 5.40 | 62.73 | 3.28 | 0.8%DIO |
| M-1F/ PC_71_BM | 0.91 | 5.14 | 55.71 | 2.60 | 1.5%DIO |
| M-1F/ PC_71_BM | 0.92 | 1.06 | 45.30 | 0.44 | None |
| M-2F/ PC_71_BM | 0.90 | 0.47 | 37.58 | 0.16 | \| None \| \| --- \| |
| M-2F/ PC_71_BM | 0.93 | 1.80 | 57.65 | 0.97 | 1.5%DIO |

**Table S2.** Device performance of M-0F, M-1F and M-2F with PBDB-T as donor.

| Donor/Acceptor | *Voc*  (V) | *Jsc*  (mA cm^-2^) | *FF*  (%) | *PCE*  (%) | additive |
| --- | --- | --- | --- | --- | --- |
| PBDB-T/ M-0F | 0.99 | 1.88 | 30.95 | 0.58 | 1%CN |
| PBDB-T/ M-0F | 0.97 | 1.71 | 31.58 | 0.52 | None |
| PBDB-T/ M-1F | 0.98 | 4.62 | 40.84 | 1.85 | 1%CN |
| PBDB-T/ M-1F | 0.98 | 4.02 | 38.24 | 1.50 | None |
| PBDB-T/ M-2F | 0.94 | 5.85 | 39.83 | 2.18 | None |
| PBDB-T/ M-2F | 0.95 | 6.25 | 44.54 | 2.65 | 1%CN |
| PBDB-T/ M-2F | 0.94 | 5.29 | 39.41 | 1.97 | 1.25%CN |
| PBDB-T/ M-2F | 0.92 | 5.58 | 38.26 | 1.96 | \| 1.5%CN \| \| --- \| |

Figure S1 (a) PL spectra of PC_71_BM, M-0F, M-1F, M-2F, M-0F/PC_71_BM, M-1F/PC_71_BM and M-2F/PC_71_BM films; the samples were excited at 650 nm (b) PL spectra of PBDB-T, M-0F, M-1F, M-2F, M-0F/ PBDB-T, M-1F/ PBDB-T and M-2F/ PBDB-T films; the samples were excited at 740 nm.

Figure S2 2D GIWAXS patterns of pristine M-0F (a), pristine M-1F（b）pristine M-2F (c) and pristine PBDB-T (d)film. Out-of-plane (e) and in-plane (f) cuts of the corresponding 2D GIWAXS patterns.

Figure S3 SCLC curves of pristine M-0F, M-1F and M-2F films and their PC_71_BM or PBDB-T blended films for hole mobility (a, b, c) and electron mobility (d, e, f).

**PDT2FBT-CHO**

^1^H-NMR


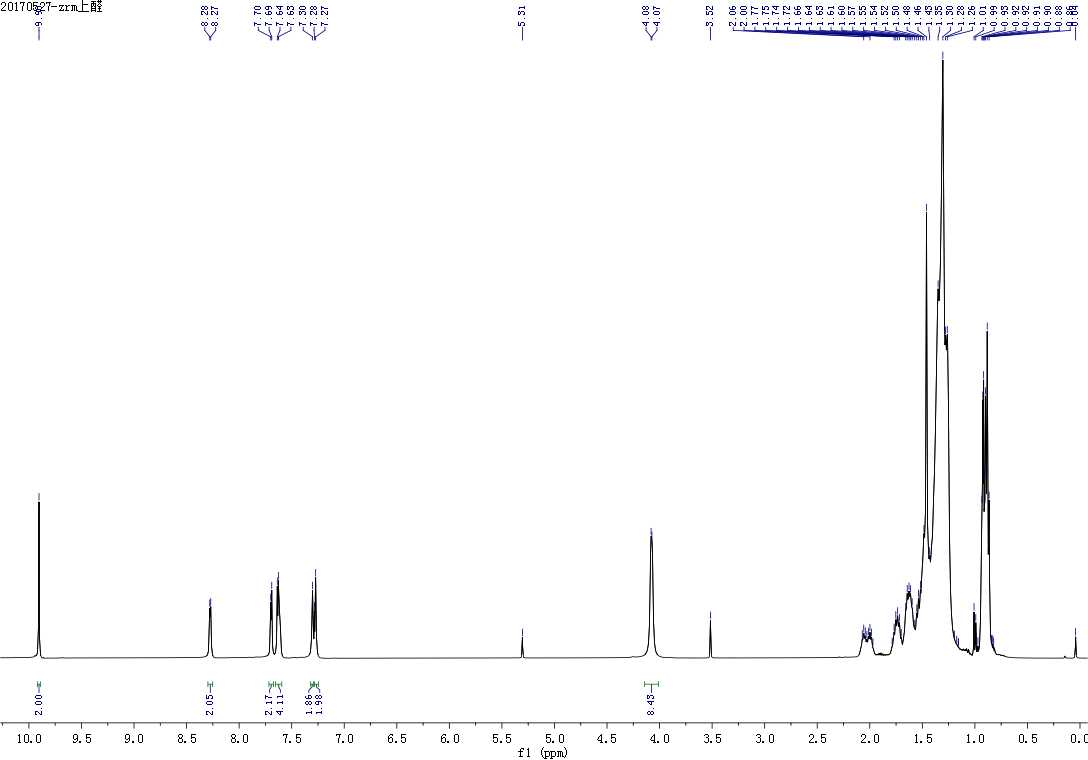


**M-OF**

^1^H-NMR

**
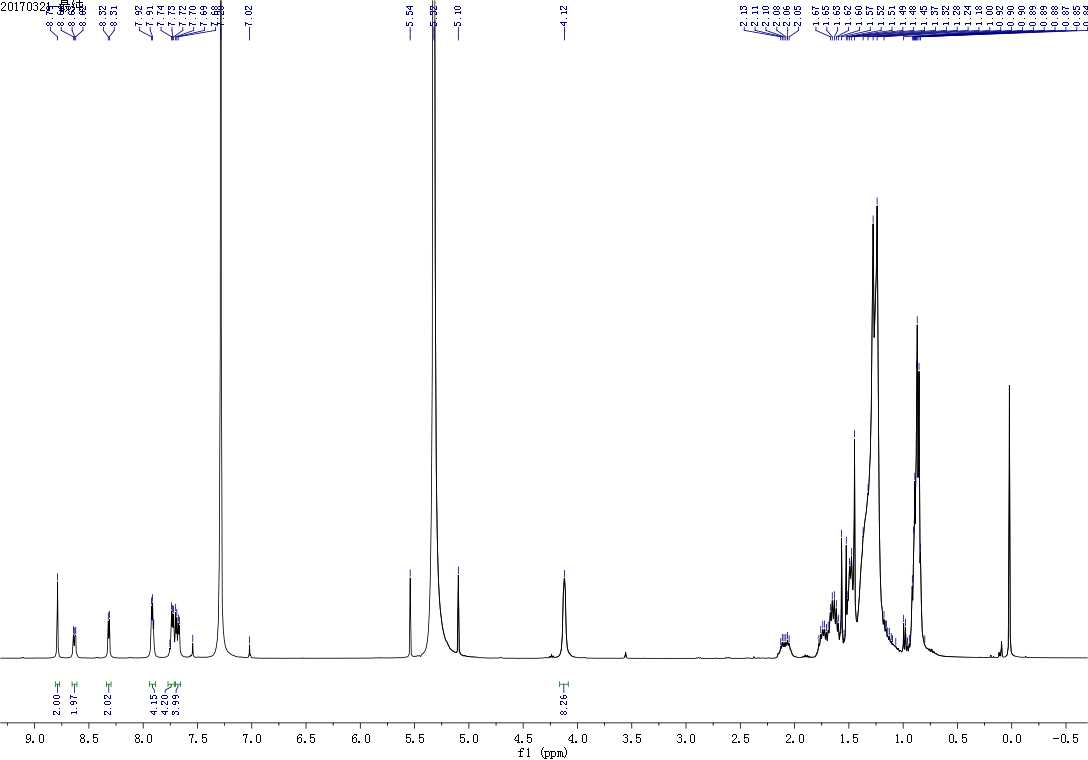
**

**M-1F**

^1^H-NMR

**
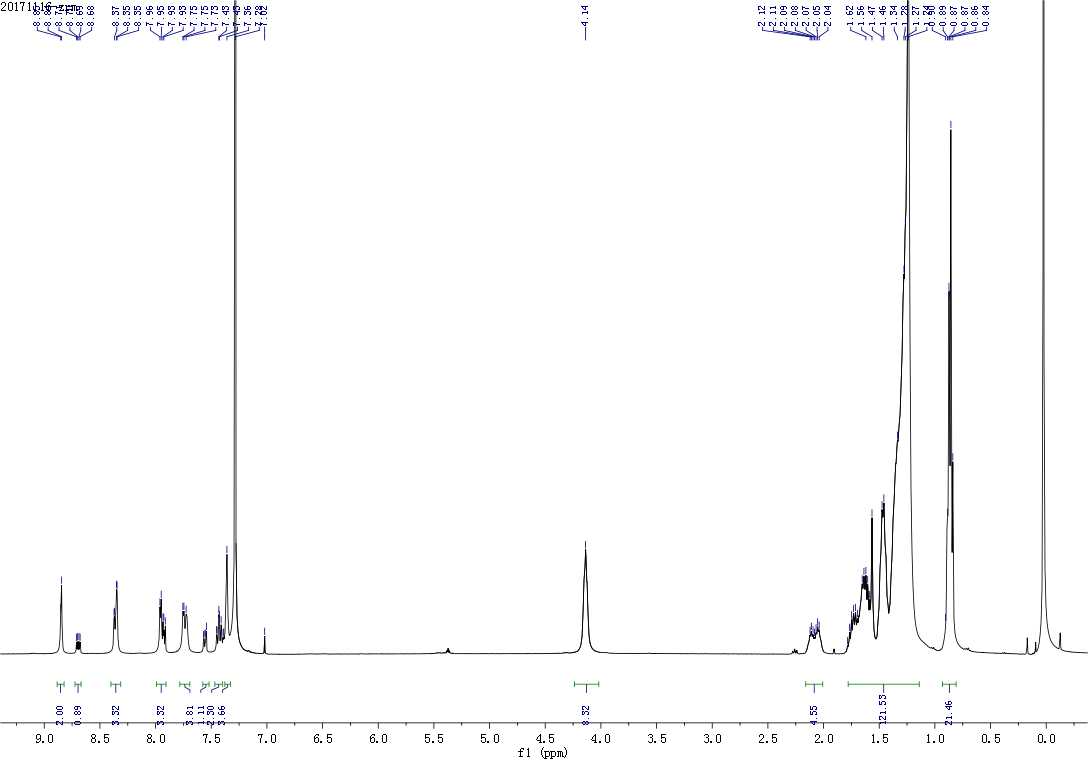
**

**M-2F**

^1^H-NMR


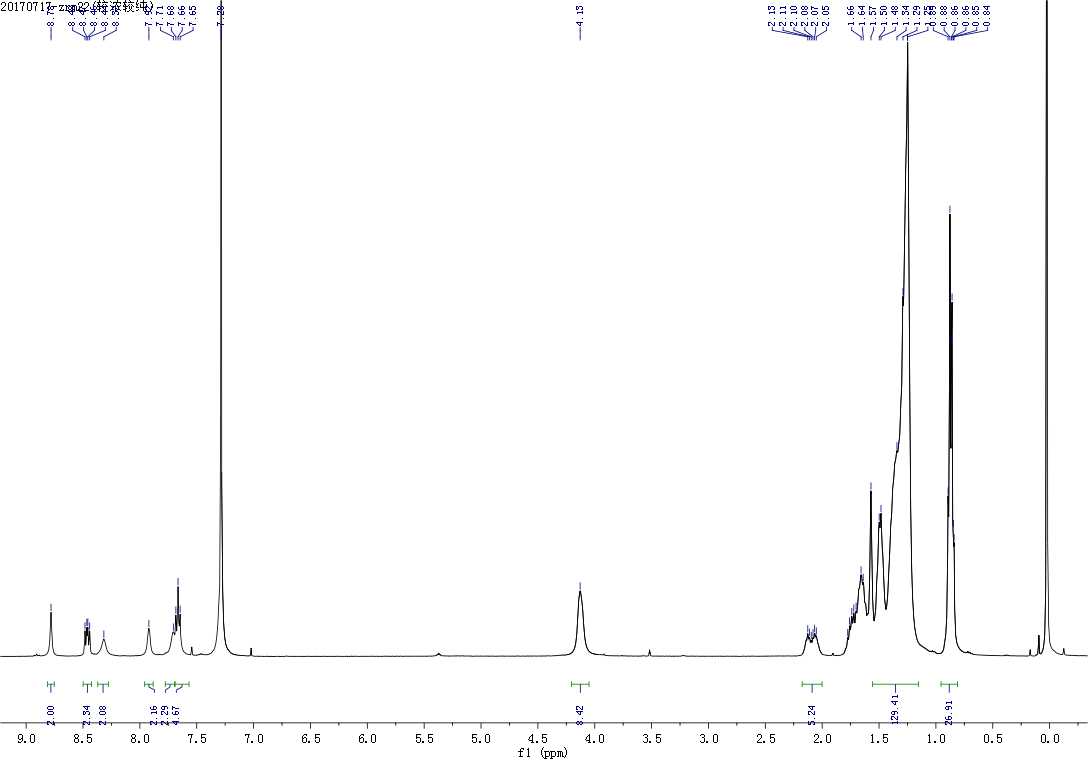

Supplement: Supplementary file 1 [file Table_1.DOCX]
